# Effectiveness and cost-effectiveness of an intensive and abbreviated individualized smoking cessation program delivered by pharmacists: A pragmatic, mixed-method, randomized trial

**DOI:** 10.1177/17151635221128263

**Published:** 2022-10-12

**Authors:** Leslie C.E. Phillips, Hai Nguyen, Terri L. Genge, W. Joy Maddigan

**Affiliations:** School of Pharmacy; Faculty of Medicine, Memorial University, St. John’s, NL; School of Pharmacy; School of Pharmacy; School of Nursing

## Abstract

**Background:**

Tobacco use is the leading preventable cause of morbidity and mortality in Canada. Smoking cessation programs (SCPs) that are effective, cost-effective and widely available are needed to help smokers quit. Pharmacists are uniquely positioned to provide such services. This study compares the abstinence rates between 2 pharmacist-led SCPs and the cost-effectiveness between these and a comparator group. The study was conducted in St. John’s, Newfoundland and Labrador.

**Methods:**

This pragmatic, mixed-method trial randomized smokers to either an existing intensive SCP or a new abbreviated SCP designed for community pharmacies. The primary outcome was 6-month abstinence rates. Cost-effectiveness was determined using abstinence rates for the SCPs and a comparator group. Incremental costs per additional quit were calculated for the trial duration, and incremental costs per life-year gained were estimated over a lifetime.

**Results:**

Quit rates for the SCPs were 36% (intensive) and 22% (abbreviated) (*p* = 0.199). Incremental costs per life-year gained for the SCPs were $1576 (intensive) and $1836 (abbreviated). The incremental costs per additional quit, relative to the comparator group, for the SCPs were $1217 (intensive) and $1420 (abbreviated).

**Discussion:**

Both SCPs helped smokers quit, and quit rates exceeded those reported for a comparator group that included a general population of adult smokers (~7%). The incremental costs per additional quit for both SCPs compare favourably to those reported for other initiatives such as quit lines and hospital-based interventions.

**Conclusion:**

Pharmacist-led smoking cessation programs are effective and highly cost-effective. Widespread implementation, facilitated by remuneration, has potential to lower smoking prevalence and associated costs and harms.

## Introduction

Tobacco use is the leading preventable cause of morbidity and mortality in Canada. Half of tobacco users die prematurely from smoking, and for every death, 30 more tobacco users struggle with related illness, including respiratory and cardiovascular diseases and cancers.^1-3^

**Knowledge Into Practice**

- Smoking is the leading preventable cause of morbidity and mortality in Canada, resulting in 50,000 deaths per year and approximately 1 million individuals who are currently dealing with chronic smoking-related illness.
- The prevalence of current smokers aged 15 and over in Canada is about 13% and has not changed significantly in the last several years.
- Pharmacists are uniquely positioned to deliver evidence-based smoking cessation services that are highly effective in getting smokers to quit as well as being highly cost-effective.

**Mise En Pratique Des Connaissances**

- Le tabagisme est la principale cause évitable de morbidité et de mortalité au Canada, entraînant 50 000 décès par année, et environ 1 million de personnes sont actuellement atteintes d’une maladie chronique liée au tabagisme.
- La prévalence actuelle des fumeurs âgés de 15 ans et plus au Canada est d’environ 13 % et n’a pas significativement changé au cours des dernières années.
- Les pharmaciens sont particulièrement bien placés pour fournir des services de désaccoutumance au tabac fondés sur des données probantes et très efficaces pour inciter les fumeurs à cesser de fumer, tout en étant très rentables.

Tobacco use is responsible for $6.5 billion in direct health care costs, $16.2 billion in total economic costs and an estimated 1 in 5 deaths in Canada.^4^ The Conference Board of Canada recommended expansion of smoking cessation programs (SCPs) in community pharmacies to obtain significant health benefits and cost savings. The report estimated that for every dollar spent on smoking cessation, a return on investment of $9.10 by 2035 could be realized.^5^

The prevalence of smoking has not changed significantly in the past decade. In 2019, 12% of Canadians aged 15 years or older identified as current smokers.^6^ Meeting Health Canada’s targeted goal of reducing tobacco use to less than 5% by 2035 (known as “5 by 35”) will require a multifaceted approach to prevent new smokers and help current smokers to quit.^7^

Effectiveness and reach are key components of population-based smoking cessation strategies. Pharmacists are uniquely positioned to systematically identify and engage smokers in quitting. Most Canadians live within 5 km of a pharmacy.^8^ Most pharmacies offer extended hours of operation, and pharmacists see their patients approximately 14 times per year.^9^ Smokers report that support from a pharmacist would enhance their ability to quit.^10^ Pharmacists are trained to provide smoking cessation and are the primary provider of quit medications. In the majority of provinces, pharmacists have full prescriptive authority for quit medications. Pharmacists in all provinces except British Columbia have prescriptive authority for smoking cessation, although some require additional training or certification. Pharmacists in Yukon, Northwest Territories and Nunavut do not appear to have prescriptive authority to date. Evidence suggests that pharmacists have an important role in tobacco cessation, including counselling, prescription and monitoring of quit medications and follow-up.^11-27^

A small number of studies examined the effectiveness of pharmacist-led SCPs and varied widely in intervention and design. Few were Canadian.^14,19,26^ Some used randomized controlled designs to evaluate effectiveness,^15-17,20,26^ but few assessed cost-effectiveness.^23,24^ The variation in definitions, timelines and methods to measure abstinence makes comparisons difficult. Many studies provided free or subsidized nicotine quit medications or incentives to participants for completing assessments.^10-12,14-16,26,27^

The aim of this study was to compare the effectiveness and cost-effectiveness of 2 smoking cessation programs in Newfoundland and Labrador to determine whether the evidence supports widespread implementation of such pharmacist-led initiatives. The study objectives were (1) to compare the abstinence rates between the 2 SCPs and (2) to determine the cost-effectiveness of the SCPs relative to each other and a literature-based comparator group.

## Methods

### Design

This article focuses on the quantitative component of a randomized, mixed-methods trial that explored the effectiveness and cost-effectiveness of 2 pharmacist-led SCPs. Qualitative methods were used to assess participants’ thoughts and feelings about the SCPs and will be reported separately. The Health Research Ethics Board of Newfoundland and Labrador approved the study (#20181188).

### Participants

The study was conducted at the Medication Therapy Services Clinic (MTSC) in St. John’s, Newfoundland and Labrador. The MTSC is a licensed pharmacy offering pharmacist-led cognitive services. Individuals aged 19 or older, currently using tobacco products and who self-referred or were referred by a health care professional to the SCP between September 2018 and March 2020 were invited to participate. Eligible smokers who provided written consent were enrolled and randomized to 1 of the 2 study arms, using a computer-generated randomization code. No monetary incentives for participation or free quit medications were provided. A prestudy sample size calculation based upon 2 active treatment arms and a control “usual care” group indicated that a sample size of 50 participants per group would be required. Because of recruitment issues, a literature-based comparator group was chosen in order to recruit as many smokers as possible into the active treatment groups. The protocol was modified to reflect this change.

### Interventions

#### Intensive SCP

Operating since 2016, the intensive SCP provides individualized support based upon the smokers’ needs. The first 2 weekly sessions were “pre-quit” visits, during which an individualized plan was prepared. The remaining follow-ups were typically post-quit, shorter in duration and becoming less frequent over time. The duration and frequency of follow-up in the intensive program were variable and left to pharmacist discretion.

#### Abbreviated SPC

The abbreviated SCP was designed by the MTSC and community pharmacists. It was a condensed version, developed for implementation in busy pharmacies. As such, the abbreviated SCP followed a set schedule, including 2 shorter pre-quit visits and fewer and shorter follow-ups. Appendix 1 (available in the online version of this article) shows a standardized timeline comparing the 2 SCPs.

Both SCPs followed best practice guidelines for cessation while respecting the smokers’ autonomy to decide on their care.^28^ While in-person visits were preferred, virtual contact was available. A quit medication prescription was provided if appropriate. Smoking status and quit medication adherence, effectiveness and tolerability were assessed and managed at each follow-up. While both programs encouraged a set quit date within 2 to 4 weeks of the first visit, a reduce-to-quit (RTQ) strategy characterized by gradual reduction in the amount smoked with intention to eventually quit was supported. Each program had a customized set of tools for assessment and follow-up. Three attempts were made to contact any no-shows, but if unsuccessful, participants were deemed lost to follow-up.

### Outcomes

Both SCPs were compared with respect to the following outcomes:

#### Primary outcomes

The primary outcome for both groups was self-reported, 7-day point prevalence abstinence rates at 6 months. Abstinence was assessed at every visit and reported using a data collection tool at 3 and 6 months starting from the participant’s quit day or start of RTQ. Individuals who were lost to follow-up before the 3- or 6**-**month data collection points were deemed to be smokers for the purposes of analysis.

#### Secondary outcomes

##### Other abstinence measurements

Prolonged abstinence (“Other than the first 2 weeks after quitting, have you smoked, even a puff?”) and continuous abstinence (“Did you at any time experience a slip or smoke even a puff after you quit?”) were captured to facilitate comparisons with other studies. Because early dropouts (usually before planned quit days) are typically high in SCPs, we also reported abstinence rates that excluded participants who dropped out after the first and second pre-quit visits as secondary outcomes, to allow examination of the impact of early dropouts on quit rates.^11^

##### Readiness scores

During the initial visit, participants completed a 2-question Readiness Ruler measuring the importance of quitting and the participants’ confidence in their ability to quit.^29^ Questions were scored on a scale of 1 to 10, with 1 being least important/confident and 10 representing most important/confident.

##### Cost and cost-effectiveness

We compared the cost-effectiveness of 3 strategies for smoking cessation: (1) the intensive SCP, (2) the abbreviated SCP, and (3) a literature-based comparator group. An abstinence rate of 7% for our comparator group was based on reported 6-month quit rates in a general population of adult smokers in Canada.^30-32^

##### Other

Baseline information was collected, including sociodemographic data, smoking history, medical history and current medication. We documented the participants’ journey during the study including quit methods and medications used and number of visits and hours spent in the SCP. Adherence to quit medication was assessed using patient report, and concerns were checked against refill frequencies using the province’s online pharmacy database.

### Statistical Methods

#### Quantitative analysis

SPSS version 27 was used to analyze demographic characteristics and smoking-related outcomes. For nominal and ordinal variables, cross tabulations using the χ^2^ test determined the statistical significance of relationships between variables, and results were presented using frequencies and percentages. Variables that consisted of interval or ratio levels of measurements were analyzed using the 2-samples *t*-test to determine relationships between the 2 groups under study. These findings were presented using means and standard deviations. Alpha was set at 0.05.

#### Cost-effectiveness analysis

A cost-effectiveness analysis was completed using the estimated average cost of intervention per participant, incremental cost per additional quit and incremental cost per life-year gained for both SCP programs.

### Costs

Costs were estimated using the health system perspective. Intervention costs included cost of the pharmacist’s time and cost of quit medications (Table 1). To estimate personnel costs, the pharmacist’s time was valued at $50 per hour (personal communication, Pharmacists Association of Newfoundland and Labrador). The average time spent by pharmacists per participant was 4.2 hours for the intensive program and 1.4 hours for the abbreviated program. These numbers were calculated from the total time spent with all participants, including those lost to attrition.

**Table 1:** Cost inputs

Parameter	Value, in $
Average hourly wage of pharmacist	50
Medication costs (3-month treatment)*	
Long-acting NRT	230^†^
Short-acting NRT	55^‡^
Bupropion SR 150 mg twice daily	75
Varenicline 1 mg twice daily	210

NRT, nicotine replacement therapy.

* Includes $10 monthly dispensing fee where applicable.

† Cost of nicotine patch.

‡ Calculated as one-sixth of average monotherapy cost of nicotine gum or inhaler. Most participants used a short-acting NRT when needed versus regularly, often in combination with a NRT patch. As such, doses and frequency of use were much lower than the published costs based on regular use as a monotherapy (e.g., regular use of a NRT inhaler as monotherapy suggests an average of 6 cartridges per day). However, when used as needed, smokers typically use less than 1 cartridge a day.

Quit medication costs for the 2 intervention groups were also estimated. For each participant, the type and dosing schedule (regular or prn [as needed]) of any prescribed quit medication was determined. The cost of each medication was calculated as the cost of a 3-month supply of a usual dose of that medication based upon a regular or prn dosing schedule.^33,34^ Next, the average cost of medications across all participants in each intervention group for whom quit medication was prescribed was calculated.

No personnel costs were incurred for the hypothetical comparator group. However, data from national surveys indicate that 39.1% of smokers use quit medications on their own. Thus, our analysis included the cost of quit medications for 39.1% of the comparator group.^6^ The average cost of quit medications reported by participants across the 2 intervention groups was used to estimate the per capita cost of quit medications in the comparator group.

### Effectiveness

Effectiveness was measured by (1) the number of participants who reported having quit smoking at 6 months and (2) gains in years of life associated with quitting. All participants assigned to each intervention group were included in the analysis.^35^ Participants lost to follow-up were assumed to have not quit.

#### Cost-effectiveness analysis

The cost-effectiveness of the intensive SCP, the abbreviated SCP and a comparator group was measured in terms of incremental cost-effectiveness ratios (ICER), estimated as the ratio of the difference in cost of 2 strategies to the difference in effectiveness. Two incremental cost-effectiveness measures were calculated: (1) incremental cost per additional quit and (2) incremental cost per life-year gained. Incremental cost per additional quit was calculated for the 6-month trial duration. Incremental cost per life-year gained was estimated over a lifetime horizon.

The cost per additional quit was estimated by using the estimated costs and abstinence rates observed in each intervention group at the 6-month follow-up. To obtain cost per life-year gained, long-term life-year gains associated with abstinence rates observed in the trial were projected using an approach proposed by Stapleton and West^36^ that has been adopted by recent, high-quality studies.^37^ Stapleton and West estimated life-years gained attributable to a smoking cessation intervention for alternative effect sizes of the intervention assuming that individuals gain 6 to 9 years of life by quitting smoking between age 35 to 54 years. Stapleton and West discounted these gains using a discount rate of 3.5%, as gains in life-years realized in the future are worth less than immediate gains. Their calculations also account for the possibility of cessation in the future in the quit-on-your-own group and potential relapse after final follow-up for intervention participants. As a result, discounted life-year gains are a more conservative approach than the undiscounted life-year gains of 6 to 10 years often reported in the literature.

## Results

### Participant flow

**Figure 1:**
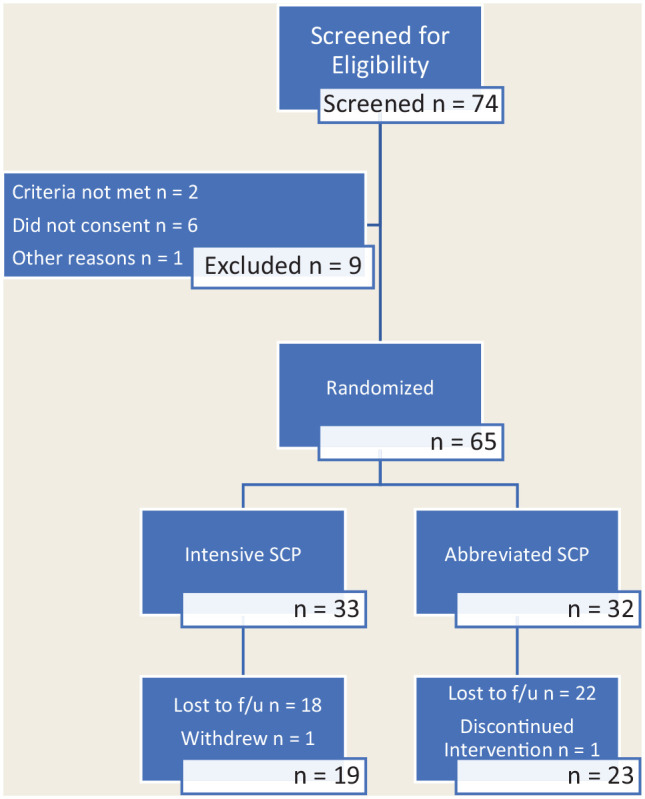
CONSORT flow diagram

Participant flow through the study is highlighted in Figure 1.

### Baseline characteristics

There were no statistically significant differences in the baseline characteristics of the 2 groups (Table 2). Most participants were middle age, Caucasian, with an approximate 33 pack-year history. A high prevalence of psychiatric, cardiovascular and respiratory disease was reported. Most made multiple prior attempts to cut back or quit, using primarily cold turkey, e-cigarettes, or varenicline. All smoked tobacco cigarettes.

**Table 2:** Demographic characteristics and tobacco use at baseline

	Control (Intensive) (*n* = 33)	Abbreviated (*n* = 32)	*p* value
**Baseline demographics**			
Age, years, mean ± SD	50.9 ± 12.21	52.50 ± 11.99	0.591
Gender, n (%) male	17 (51.5)	10 (31.3)	0.097
Total number of medical conditions reported, mean ± SD	1.79 ± 1.19	2.19 ± 1.28	0.198
Classification of medical conditions, n (%)			
Psychiatric	18 (54.5)	17 (53.1)	0.909
Cardiovascular	14 (42.4)	13 (40.6)	0.883
Respiratory	8 (24.2)	9 (28.1)	0.722
Neurologic	5 (15.2)	5 (15.8)	0.958
Dermatologic	4 (12.1)	7 (21.9)	0.294
Cancer	2 (6.1)	5 (15.6)	0.214
Musculoskeletal	3 (9.1)	2 (6.3)	0.667
Gastrointestinal	2 (6.1)	5 (15.6)	0.214
Endocrine	3 (9.1)	7 (21.9)	0.153
**Tobacco use history**			
Age started smoking, years, mean ± SD	15.26 ± 3.54	17.06 ± 4.54	0.086
Number of years smoking, mean ± SD	32.13 ± 13.81	33.91 ± 12.22	0.601
Tried cutting back tobacco use in past, n (%)	28 (93.3)	31 (100.0)	0.144
Estimated number of quit attempts over lifetime, mean ± SD	9.59 ± 18.95	18.72 ± 38.65	0.279
**Past quits, methods tried, n (%)**			
Cold turkey	16 (51.6)	18 (60.0)	0.510
Hypnosis	1 (3.2)	4 (13.3)	0.150
Acupuncture	0 (0.0)	2 (6.7)	0.144
Electronic cigarettes	8 (25.8)	6 (20.0)	0.590
Bupropion	5 (16.1)	9 (30.0)	0.198
Varenicline	12 (38.7)	14 (46.7)	0.530
Laser	1 (3.2)	0 (0.0)	0.321
**Information related to current quit/RTQ attempt**			
Smoking status at baseline, n (%)			0.219
Cigarettes only	29 (90.6)	25 (78.1)	
Cigarettes + e-cigarettes	1 (3.1)	4 (12.5)	
Cigarettes + cannabis	2 (6.3)	1 (3.1)	
Cigarettes + cigarillos	0 (0.0)	2 (6.3)	
Cigarettes smoked per day	19.67 (9.65)	20.34 (10.86)	0.791
Chose RTQ method with no quit date, n (%)	10 (30.3)	5 (15.6)	0.160
Chose a set quit date, n (%)	26 (78.8)	21 (65.6)	0.236
Quit on chosen quit date, n (%)	11 (45.8)	11 (73.3)	0.092
Initial pharmacotherapy, n (%)			0.418
Varenicline only	13 (39.4)	6 (18.8)	
Varenicline + NRT prn	1 (3.0)	1 (3.1)	
NRT patch only	3 (9.1)	4 (12.5)	
NRT patch + NRT prn	7 (21.2)	10 (31.3)	
Bupropion only	1 (3.0)	0 (0.0)	
Bupropion + NRT prn	1 (3.0)	0 (0.0)	
Short-acting NRT prn only	4 (12.1)	3 (9.4)	
No medications	2 (6.1)	7 (21.9)	
Other combination	1 (3.0)	1 (3.1)	
Documented chart note of significant adherence issues regarding quit medication, n (%)	12 (54.5)	13 (72.2)	0.251
Pre-quit Readiness Ruler—Importance Score, mean ± SD	9.18 ± 1.15	9.13 ± 1.23	0.874
Pre-quit Readiness Ruler—Confidence Score, mean ± SD	6.39 ± 2.34	6.44 ± 2.30	0.935

NRT, nicotine replacement therapy; RTQ, reduce-to-quit. For additional baseline data, refer to Appendix 2 (available in the online version of this article).

Most participants picked a quit date within 2 to 4 weeks from the initial visit and opted to use quit medication. Varenicline and combination nicotine replacement therapy (NRT) were most common. A few chose RTQ with no set quit date. At visit 1, participants in both groups expressed quitting as a priority but were less confident in their ability to quit.

### Abstinence rates

**Figure 2:**
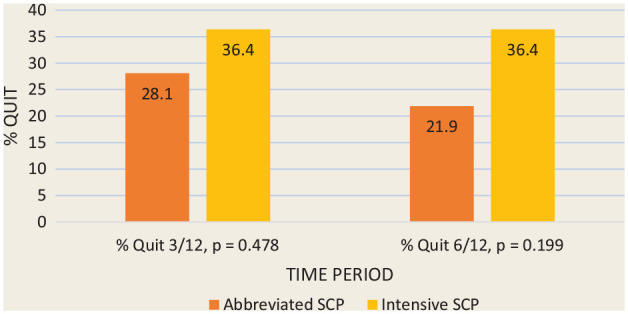
Primary outcome: 7-day point prevalence abstinence rates (%) at 3 and 6 months for the 2 study groups (*n* = 65)

Figure 2 depicts the 7-day point prevalence reported abstinence at 3 and 6 months using intention-to-treat principles. The 6-month quit rates were 36% and 22% in the intensive and abbreviated SCPs, respectively. A statistically significant difference was not found. This compares to an estimated quit rate of about 7% among the general population of adult smokers.^32^ Abstinence at 3 months predicted abstinence at 6 months. Ninety-eight percent of individuals who were quit at 3 months were also quit at 6 months (*p* = 0.000).

**Figure 3:**
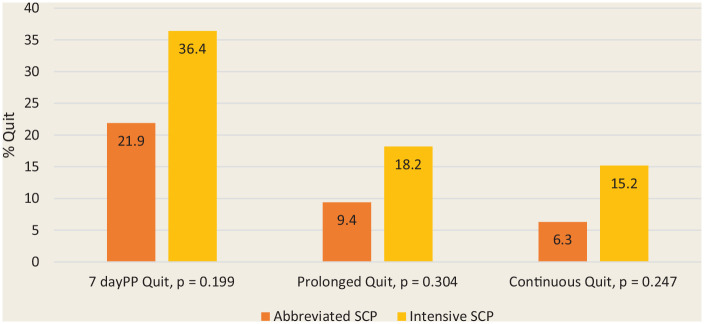
The 7-day point prevalence, prolonged and continuous abstinence rates (%) at 6 months for the intensive and abbreviated programs (*n* = 65)

Abstinence rates at 6 months using more stringent definitions of abstinence are shown in Figure 3. Prolonged abstinence allows a 2-week grace period following the quit day, but any slips beyond that would be classified as “not quit.” Continuous abstinence is more stringent, requiring that the smoker remain quit throughout.

**Figure 4:**
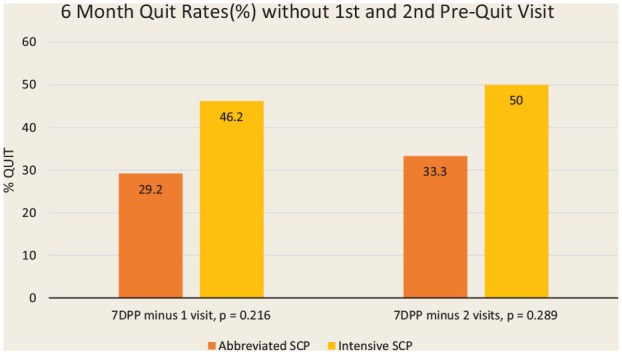
The 7-day point prevalence abstinence rates (%) at 6 months for the intensive and abbreviated programs *Excluding those only staying for first pre-quit visit and only staying for first 2 pre-quit visits.

For Figures 2 and 3, all participants lost to follow-up were considered smokers, providing a more conservative measure of abstinence. In this study, 50% of participants did not return after the first 2 pre-quit visits. Most of these dropouts occurred following the first visit when the quit plan was still being developed. A secondary, modified intention-to-treat analysis of 7-day point prevalence quit rates at 6 months, excluding individuals who only stayed for the first visit and those who only stayed for the first 2 visits, was completed (Figure 4). Participants who remained in the study after this time but were subsequently lost to follow-up were included in the analysis as “not quit.” The 7-day point prevalence abstinence rates at 6 months rose from 36% (all participants) to 46% (excluded those who only came to first visit) to 50% (excluded those who only came for first 2 visits) in the intensive SCP and from 22% to 29% to 33%, respectively, in the abbreviated program. Participants who stayed beyond 2 visits were more likely to quit.

### RTQ participants

Fifteen participants opted for a RTQ strategy with no set quit date. Of these, 10 participants (5 male) were in the intensive SCP and 5 participants (2 male) were in the abbreviated SCP. One participant withdrew from the study to receive sample NRT and subsequently quit smoking. Eleven participants were lost to follow-up by 6 months. Three participants had 6-month data. Two participants quit and 1 participant, who had reduced by 70%, relapsed.

### Cost and cost-effectiveness

#### Cost estimates

Intervention costs for the 2 SCPs and a comparator group are shown in Table 3.

**Table 3:** Intervention costs (per person) during 6-month period

	**Intensive program, $**	**Abbreviated program, $**	**Comparator, $**
Personnel costs (A)	210	70	0
Average medication costs (B)*	245	245	102
Total costs per participant (A + B)	455	315	102

* The cost of a 3-month supply is assigned to each medication type. For comparator group, it is assumed that 39.1% of individuals use quit medications in the absence of a smoking cessation intervention.^4^

#### Cost-effectiveness estimates

Abstinence rates of 36% (intensive SCP), 22% (abbreviated SCP) and 7% (comparator group) were used for this analysis. Results are presented in Table 4. Panel A shows that compared with the comparator group, the abbreviated SCP cost $21,300 more but resulted in 15 additional quits per 100 participants. The incremental cost per additional quit was $1420. Applying Stapleton and West’s approach, the 15 additional quits translated into 11.6 additional discounted life-years gained, yielding an ICER of $1836 per life-year gained.

**Table 4:** Cost-effectiveness analysis

	Total cost per 100 persons, $	Incremental cost per 100 persons, $	Total quits per 100 persons	Incremental quits per 100 persons	Incremental life-years gained per 100 persons	Incremental cost per additional quit, $	Incremental cost per life-year gained, $
*Panel A: All strategies*							
Comparator group	10,200	—	7	—	—	—	—
Abbreviated program	31,500	21,300	22	15	11.6	1420	1836
Intensive program	45,500	14,000	36	14	10.8	1000	1296
*Panel B: Intensive program vs comparator group*							
Comparator group	10,200	—	7	—	—	—	—
Intensive program	45,500	35,300	36	29	22.4	1217	1576

In Panel A, incremental costs, quits and life-years gained are compared with the next less costly strategy. That is, the abbreviated program is compared with the comparator group and the intensive program is compared with the abbreviated program. Incremental cost per additional quit was calculated for the trial duration: that is, 6 months. Incremental cost per life-year gained was estimated over a lifetime horizon.

The intensive SCP cost $14,000 (per 100 participants) more than the abbreviated SCP but resulted in 14 additional quits and 10.8 additional life-years gained. The intensive SCP had an incremental cost of $1000 per additional quit and $1296 per life-year gained relative to the abbreviated SCP. The ICERs for the abbreviated SCP (relative to the comparator group) were higher than those for the intensive SCP (relative to the abbreviated program). This implies that more quits can be achieved (and life-years gained) at a lower cost per quit per life-year with the intensive versus the abbreviated SCP. The intensive SCP was the most cost-effective intervention of the 3 strategies. Panel B in Table 4 shows that relative to the comparator group, the intensive SCP will cost $35,300 more (per 100 participants) to the health care system but also results in 29 more quits and 22.4 additional life-years gained, yielding ICERs of $1217 per additional quit and $1576 per life-year gained.

## Discussion

As health care expenditures continue to rise, assessing the effectiveness and cost-effectiveness of new interventions will allow clinicians and policy-makers to make rational decisions regarding optimal resource allocation. This is one of few studies comparing both quit rates and cost-effectiveness of pharmacist-led SCPs. In this study, both an intensive (primary care setting) SCP and an abbreviated SCP (designed for implementation in community pharmacies) were successful in helping participants quit and both were very cost-effective. Widespread implementation of the abbreviated SCP in community settings could be instrumental in helping Canada reach its “5 by 35” goal.

At 6 months, both SCPs in this study produced substantive abstinence rates of 36% (intensive SCP) and 22% (abbreviated SCP), demonstrating their effectiveness. The 6-month quit rate for a general population of adult smokers in Canada is approximately 7%.^32^ The 6- to 12-month quit rate for untreated smokers averages around 3% to 5%.^30^ Counselling and/or medication intervention increases this figure to 10% to 30%.^31^ A meta-analysis reported abstinence rates in community pharmacy-based SCPs to range from 11.6% to 36.4%.^38^

This study measured 6-month, point-prevalence abstinence rates using intention-to-treat principles as its primary outcome, but the impact of early attrition on quit rates is substantive. Preintervention attrition rates ranging from 30% to 50% have been reported in studies.^39^ The exclusion of early dropouts from analysis dramatically improves quit rates. In this study, participants who stayed for 3 or more visits had a 50% and 33% chance of quitting in the intensive and abbreviated SCPs, respectively. This supports the importance of enhanced efforts by clinicians at the outset to educate and encourage smokers to remain committed.

The cost-effectiveness analysis suggests that the costs of the SCPs yield benefits that are comparable (or even larger) than other smoking cessation interventions. Specifically, the incremental costs per additional quit for the intensive SCP and abbreviated SCP relative to the comparator group ($1217 and $1420 per additional quit, respectively) were lower than the estimates for other smoking cessation interventions such as population outreach programs, tobacco quit lines and hospital-based quit interventions, all exceeding $2000 per quit. Applying previously estimated ratios of quality-adjusted life-years gained to life-years gained (ranging between 0.7 and 1.4), the incremental cost per life-year gained ($1576 for the intensive SCP and $1836 for the abbreviated SCP) is also far lower than the conventional threshold of $50,000 per quality-adjusted life-year gained.^37,40-42^

The primary limitations of this study were a small sample size related to recruitment issues and the resultant need to resort to a historical comparator group. A large number of dropouts, limited research funding, and COVID-19–related shut-downs all contributed to recruitment challenges. A third “quit-on-your-own” control arm was viewed as desirable but subsequently deemed not feasible, and a literature-based comparator was used instead. The significant dropout rate and inability to achieve our sample size likely translated into a type 2 statistical error, underpowering the study’s ability to detect any significant differences between the 2 groups.

Measurement of abstinence rates using only self-report has been criticized. Biochemical validation via carbon monoxide, or cotinine (which also picks up NRT), has been recommended to confirm abstinence.^43-45^ No method is ideal or confirms long-term abstinence. We chose self-report as the least expensive, least invasive and most pragmatic approach for a community pharmacy setting. While smokers may overestimate their self-reported successes out of a desire to please or pressure to quit, these same motivations might also cause them to temporarily abstain in order to do well on scheduled biochemical validation tests.^46^

This study was conducted in Newfoundland and Labrador, which has the highest provincial smoking prevalence rates.^6^ It is important to note that while the study population identified as Caucasian, other ethnicities were not excluded intentionally. This merely reflects the overall population on the island. Participants were not questioned and did not self-identify as Indigenous or 2SLGBTQIA+. Approximately half identified as having a psychiatric condition, the majority of which were anxiety or mood disorders.

The main strength of this study was its assessment of both effectiveness and cost-effectiveness. Intention-to-treat principles were followed for the primary outcome. The provision of quit medication samples that could further bias success rates was prohibited. Community pharmacists contributed to the design of the abbreviated SCP to facilitate its implementation in busy community settings. Continuous, prolonged and point-prevalence abstinence rates at 3 and 6 months were captured to facilitate comparison with other studies.

## Conclusion

Community pharmacists are an accessible, underutilized and cost-effective resource to provide an array of public health services, including vaccinations and smoking cessation.^5,7,8,9,18^ Smoking cessation is the most powerful preventive intervention a clinician can make. For every 2 smokers who quit, 1 tobacco-related death is prevented.^47^

Two pharmacist-delivered SCPs were examined in this study. Both were effective, with self-reported, 7-day point prevalence abstinence rates at 6 months of 36% and 22% for the intensive and abbreviated SCPs, respectively. Both were highly cost-effective, even when compared with traditionally offered services. Widespread implementation facilitated by adequate reimbursement of pharmacist-led SCPs can effectively and cost-effectively affect smoking rates and associated morbidity and mortality and help us reach “5 by 35.” ■

## Supplemental Material

sj-pdf-1-cph-10.1177_17151635221128263 – Supplemental material for Effectiveness and cost-effectiveness of an intensive and abbreviated individualized smoking cessation program delivered by pharmacists: A pragmatic, mixed-method, randomized trial Click here for additional data file. Supplemental material, sj-pdf-1-cph-10.1177_17151635221128263 for Effectiveness and cost-effectiveness of an intensive and abbreviated individualized smoking cessation program delivered by pharmacists: A pragmatic, mixed-method, randomized trial by Leslie C.E. Phillips, Hai Nguyen, Terri L. Genge and W. Joy Maddigan in Canadian Pharmacists Journal / Revue des Pharmaciens du Canada

sj-pdf-2-cph-10.1177_17151635221128263 – Supplemental material for Effectiveness and cost-effectiveness of an intensive and abbreviated individualized smoking cessation program delivered by pharmacists: A pragmatic, mixed-method, randomized trial Click here for additional data file. Supplemental material, sj-pdf-2-cph-10.1177_17151635221128263 for Effectiveness and cost-effectiveness of an intensive and abbreviated individualized smoking cessation program delivered by pharmacists: A pragmatic, mixed-method, randomized trial by Leslie C.E. Phillips, Hai Nguyen, Terri L. Genge and W. Joy Maddigan in Canadian Pharmacists Journal / Revue des Pharmaciens du Canada
