# Supplementary material for: Effectiveness and cost-effectiveness of an intensive and abbreviated individualized smoking cessation program delivered by pharmacists: A pragmatic, mixed-method, randomized trial
Source: Can Pharm J (Ott). 2022 Oct 12;155(6):334–44. doi: 10.1177/17151635221128263 (PMC9647399; doi:10.1177/17151635221128263)
Supplement: sj-pdf-1-cph-10.1177_17151635221128263 – Supplemental material for Effectiveness and cost-effectiveness of an intensive and abbreviated individualized smoking cessation program delivered by pharmacists: A pragmatic, mixed-method, randomized trial [file sj-pdf-1-cph-10.1177_17151635221128263.pdf]

APPENDIX 1 Intensive and abbreviated smoking cessation program content and timelines*

Visit	Intensive SCP Duration	Abbreviated SCP Duration
Pre-Quit Visit 1	1 hour<br>• Complete Readiness Ruler<br>• Discuss what they Like Vs Don't like about their tobacco use<br>• Determine Reasons for Quitting<br>• Assign Pre-Quit Log<br>• Current Smoking Status<br>• Detailed Smoking History<br>• Current Medications<br>• Medical Conditions<br>• Caffeine, Alcohol, Cannabis Use and relationship to tobacco use<br>• Review Treatment Options and Medication Coverage	30 minutes<br>• Complete Readiness Ruler<br>• Current Smoking Status<br>• Brief Smoking History- what quit medications were tried and results<br>• Current Medications<br>• Medical Conditions<br>• Caffeine, Alcohol, Cannabis Use and relationship to tobacco use<br>• Review Treatment Options and Medication Coverage
Pre-Quit Visit 2	1 hour<br>• Review Pre-Quit Log and identify triggers & discuss management<br>• Review Nicotine Withdrawal symptoms and management<br>• Develop Individualized Quit Plan including Quit Methods (e,g, Reduce to Quit schedule, Set Quit Date, Medications); Prescribe quit medication and discuss use; review non-drug ways to manage cravings<br>• Discuss ways to avoid slip ups<br>• Review ways to avoid weight gain	30 minutes<br>• Review potential triggers and management<br>• Develop Individualized Quit Plan including Quit Methods (e,g, Reduce to Quit schedule, Set quit date Medications); Prescribe quit medication and discuss use, brief discussion of non-drug ways to manage cravings
Week 1 Follow-up	15-30 minutes<br>Assess/manage issues related to<br>• Adherence (use and technique)<br>• Efficacy (smoking status, severity and frequency of cravings)<br>• Tolerability	15-30 minutes<br>Assess/manage issues related to<br>• Adherence (use and technique)<br>• Efficacy (smoking status, severity and frequency of cravings)<br>• Tolerability
Week 2 Follow-up	15-30 minutes	-
Week 3 Follow-up	15-30 minutes	-
Week 4 Follow-up	15-30 minutes	-
Week 6 Follow-up	15-30 minutes	15-30 minutes
Week 8 Follow-up	15-30 minutes	-
Week 12 (3 months)	15-30 minutes	15-30 minutes
Week 16 Follow-up	15-30 minutes	-
Week 26 (6 months)	15-30 minutes	15-30 minutes

*Follow-ups in the Intensive Program were determined at the end of each visit according to client need and did not have to strictly follow the schedule above; whereas follow-ups in the Abbreviated Program adhered to the above schedule. Follow-up content through week 2-26 followed the same general process at each visit.

NOTE: Pharmacists for a Smoke Free Canada (PSFC) have free tools available on their website, including a Pharmacotherapy Treatment Algorithm, a Pre-Quit Assessment Tool (fillable pdf), a Post-Quit Follow-Up Tool (fillable pdf), and a My Quit Plan for the client (fillable pdf). The tools contain lots of tips on managing triggers, cravings/withdrawal, slips and relapses, as well as selecting medication and managing medication side effects. Other than the Pharmacotherapy Algorithm, the remaining materials were developed by the primary author in consultation with a cessation expert from PSFC, following the study and reflected experience with the study tools. These can be accessed at https://psfcnetwork.com/

Phillips LCE, et al. Effectiveness and cost-effectiveness of an intensive and abbreviated individualized smoking cessation program delivered by pharmacists: a pragmatic, mixed-method, randomized trial. Can Pharm J (Ott) 2022;155. DOI: 10.1177/17151635221128263.
