# Supplementary material for: Effectiveness and cost-effectiveness of an intensive and abbreviated individualized smoking cessation program delivered by pharmacists: A pragmatic, mixed-method, randomized trial
Source: Can Pharm J (Ott). 2022 Oct 12;155(6):334–44. doi: 10.1177/17151635221128263 (PMC9647399; doi:10.1177/17151635221128263)
Supplement: sj-pdf-2-cph-10.1177_17151635221128263 – Supplemental material for Effectiveness and cost-effectiveness of an intensive and abbreviated individualized smoking cessation program delivered by pharmacists: A pragmatic, mixed-method, randomized trial [file sj-pdf-2-cph-10.1177_17151635221128263.pdf]

**APPENDIX 2 Demographic characteristics and tobacco use at baseline**

| Baseline demographic | Control (Intensive) n=33 | Abbreviated n=32 | P value |
|---|---|---|---|
| **Age (years), mean (SD)** | 50.9 (12.21) | 52.50 (11.99) | *P* = .591 |
| **Gender, n (% Male)** | 17(51.5) | 10(31.3) | *P* = .097 |
| **Household income ($), n (%)** | | | *P* = .304 |
| Less than 20, 0000 | 4 (14.8) | 7 (24.1) | |
| 20, 000 – 49,999 | 4 (14.8) | 8 (27.6) | |
| 50, 000 – 79, 999 | 12 (44.4) | 8 (27.6) | |
| 80, 000 – 100, 000 | 2 (7.4) | 4 (13.8) | |
| More than 100, 000 | 5 (18.5) | 2 (6.9) | |
| **Medical insurance, n (%)** | | | *P = .159* |
| Private | 23 (76.7) | 18 (60.0) | |
| NLPDP*/income support | 4 (13.3) | 10 (33.3) | |
| None/cash | 3 (10.0) | 1 (3.3) | |
| Other | 0 (0.0) | 1 (3.3) | |
| **Total # medical conditions reported, mean (SD)** | 1.79 (1.19) | 2.19 (1.28) | *P* = .198 |
| **Classification of medical conditions, n (%)** | | | |
| Psychiatric | 18 (54.5) | 17 (53.1) | *P* = .909 |
| Cardiovascular | 14 (42.4) | 13 (40.6) | *P* = .883 |
| Respiratory | 8 (24.2) | 9 (28.1) | *P* = .722 |
| Neurologic | 5 (15.2) | 5 (15.8) | *P* = .958 |
| Dermatologic | 4 (12.1) | 7 (21.9) | *P* = .294 |
| Cancer | 2 (6.1) | 5 (15.6) | *P* = .214 |
| Musculoskeletal | 3 (9.1) | 2 (6.3) | *P* = .667 |

| **Baseline demographic** | **Control (Intensive) n=33** | **Abbreviated n=32** | **P value** |
|---|---|---|---|
| Gastrointestinal | 2 (6.1) | 5 (15.6) | *P* = .214 |
| Endocrine | 3 (9.1) | 7 (21.9) | *P* = .153 |
| **Tobacco use history** | | | |
| **Age started smoking(years), mean (SD)** | 15.26 (3.54) | 17.06 (4.54) | *P = .086* |
| **# years smoking, mean (SD)** | 32.13 (13.81) | 33.91 (12.22) | *P = .601* |
| **Tried cutting back tobacco use in past, n (%)** | 28 (93.3) | 31 (100.0) | *P* = .144 |
| **Estimated # quit attempts over preceding 12 months, mean (SD)** | 1.63 (2.32) | 2.10 3.17) | *P = .529* |
| **Estimated # quit attempts over lifetime, mean (SD)** | 9.59 (18.95) | 18.72 (38.65) | *P* = .279 |
| **Estimated longest time ever quit (years), mean (SD)** | 2.63 (4.92) | 1.29 (2.90) | *P = .206* |
| **Past quits- methods tried, n(%)** | | | |
| Cold turkey | 16 (51.6) | 18 (60.0) | *P* = .510 |
| Hypnosis | 1 (3.2) | 4 (13.3) | *P* = .150 |
| Acupuncture | 0 (0.0) | 2 (6.7) | *P* = .144 |
| Electronic cigarettes | 8 (25.8) | 6 (20.0) | *P* = .590 |
| Bupropion | 5 (16.1) | 9 (30.0) | *P* = .198 |
| Varenicline | 12 (38.7) | 14 (46.7) | *P* = .530 |
| Laser | 1 (3.2) | 0 (0.0) | *P* = .321 |
| **Past quits - # of methods tried, mean(SD)** | 2.19 (1.28) | 2.53 (1.17) | *P* = .264 |
| **Information related to current quit/rtq attempt** | | | |
| **Smoking status @baseline, n(%)** | | | *P = .219* |

| Baseline demographic | Control (Intensive) n=33 | Abbreviated n=32 | P value |
|---|---|---|---|
| Cigarettes only | 29 (90.6) | 25 (78.1) | |
| Cigarettes+E-Cig | 1 (3.1) | 4 (12.5) | |
| Cigarettes+Cannabis | 2 (6.3) | 1 (3.1) | |
| Cigarettes+Cigarillos | 0 (0.0) | 2 (6.3) | |
| **Cigarettes smoked per day** | 19.67 (9.65) | 20.34 (10.86) | *P = .791* |
| **Chose RTQ** method with no quit date, n (%)** | 10 (30.3) | 5 (15.6) | *P = .160* |
| **Chose a set quit date, n(%)** | 26 (78.8) | 21 (65.6) | *P = .236* |
| **Quit on chosen quit date, n(%)** | 11 (45.8) | 11 (73.3) | *P = .092* |
| **Season current quit started, n(%)** | | | *P = .161* |
| Fall | 8 (50.0) | 7 (78.8) | |
| Winter | 6 (37.5) | 0 (0.0) | |
| Spring | 2 (12.5) | 1 (11.1) | |
| Summer | 0 (0.0) | 1 (11.1) | |
| **Initial pharmacotherapy, n(%)** | | | *P = .418* |
| Varenicline – only | 13 (39.4) | 6 (18.8) | |
| Varenicline + NRTprn | 1 (3.0) | 1 (3.1) | |
| NRT Patch – only | 3 (9.1) | 4 (12.5) | |
| NRT Patch +NRTprn | 7 (21.2) | 10 (31.3) | |
| Bupropion - only | 1 (3.0) | 0 (0.0) | |
| Bupropion+NRTprn | 1 (3.0) | 0 (0.0) | |
| Short Acting NRTprn-only | 4 (12.1) | 3 (9.4) | |
| No medications | 2 (6.1) | 7 (21.9) | |
| Other combination | 1 (3.0) | 1 (3.1) | |
| **Documented chart note of significant adherence issues re quit medication, n (%)** | 12 (54.5) | 13 (72.2) | *P =.251* |
| **Pre-quit Readiness Ruler – Importance Score, mean(SD)** | 9.18 (1.15) | 9.13 (1.23) | *P = .874* |
| **Pre-quit Readiness Ruler – Confidence Score, mean(SD)** | 6.39 (2.34) | 6.44 (2.30) | *P = .935* |

*Newfoundland & Labrador Prescription Drug Program support for low-income residents

**Reduce-To-Quit

Phillips LCE, et al. Effectiveness and cost-effectiveness of an intensive and abbreviated individualized smoking cessation program delivered by pharmacists: a pragmatic, mixed-method, randomized trial. Can Pharm J (Ott) 2022;155. DOI: 10.1177/17151635221128263.
